# Circulating Hematopoietic Stem/Progenitor Cells are Associated with Coronary Stenoses in Patients with Coronary Heart Disease

**DOI:** 10.1038/s41598-018-38298-5

**Published:** 2019-02-08

**Authors:** Fu-Li Zhu, Ning Zhang, Xiao-Juan Ma, Jing Yang, Wei-Ping Sun, Yi-Qing Shen, Yu-Mei Wen, Sha-Sha Yuan, Dong Zhao, Hai-Bin Zhang, Ying-Mei Feng

**Affiliations:** 1 Department of Cardiology, Beijing LuHe Hospital, Capital Medical University, Beijing, China; 2 Beijing Key Laboratory of Diabetes Prevention and Research, Department of Endocrinology, Beijing LuHe Hospital, Capital Medical University, Beijing, China

## Abstract

Inflammatory cells in atherosclerotic plaque exclusively originate from hematopoietic stem/progenitor cells (HSPCs). In this study, we investigated whether circulating HSPCs frequency related to coronary stenosis in patients with coronary heart disease (CHD). Coronary angiography was performed in 468 participants who were recruited at Cardiology Centre in LuHe Hospital from March 2016 to May 2017. Among these subjects, 344 underwent echocardiography. Mononuclear cells isolated from peripheral blood were stained with an antibody cocktail containing anti-human CD34, anti-human lineage, anti-human CD38, and anti-human CD45RA. Lineage^−^CD38^−^CD45RA^dim^CD34^+^HSPCs were quantified by flow cytometry. CHD was defined as coronary stenosis ≥50% and the extent of CHD was further categorised by coronary stenosis ≥70%. A p < 0.0031 was regarded statistically significant by the Bonferroni correction. Circulating HSPCs frequency was 1.8-fold higher in CHD patients than non-CHD participants (p = 0.047). Multivariate-adjusted logistic analysis demonstrated that HSPCs was the only marker that was associated with the odds ratio of having mild *vs*. severe coronary stenosis (2.08 (95% CI, 1.35–3.21), p = 0.0009). Left ventricular ejection fraction was inversely correlated with HSPCs frequency and CRP in CHD patients (p < 0.05 for both). In conclusion, HSPCs frequency in circulation is intimately related to coronary stenoses in CHD patients.

## Introduction

Atherosclerosis forms the pathological basis of coronary heart disease (CHD)^1^. The initiation and progression of atherosclerosis are complicated, involving endothelial dysfunction, inflammation, oxidative stress and thrombus formation^2–4^. Risk factors such as hypercholesterolemia and diabetes enhance endothelial cell injury and inflammation and reinforce atherosclerosis progression in mice^5–7^ and humans^8,9^. Despite the implementation of various regimens having had beneficial effects in CHD patients, a large number of patients still progress to repeated stenting for revascularisation and recurrent myocardial infarction^2^. Therefore, the screening and identification of novel markers that reflect the severity of coronary stenosis are needed not only for assessing CHD but also for establishing therapeutic targets for treating this disease.

Inflammatory cell infiltration and foam cell formation are hallmarks of atherosclerosis progression^10–12^. Amplification of the inflammatory cell cascade and the production of inflammatory cytokines and chemokines accelerate coronary stenoses and plaque rupture, resulting in acute coronary syndrome and myocardial infarction. Inflammatory cells are known to be exclusively derived from hematopoietic stem/progenitor cells (HSPCs) in bone marrow. In line with the literature^13,14^, we recently reported experimental studies that showed that hypercholesterolemia and low-density lipoproteins enhance the proliferation of HSPCs and their subsequent differentiation to atherogenic neutrophils and monocytes, whereas high-density lipoproteins inhibit these processes^15,16^. In this study, we moved from analyses of mice to human subjects to confirm that HSPCs in circulation could be associated with coronary stenoses.

## Results

### Characteristics of CAD patients

**Figure 1:**
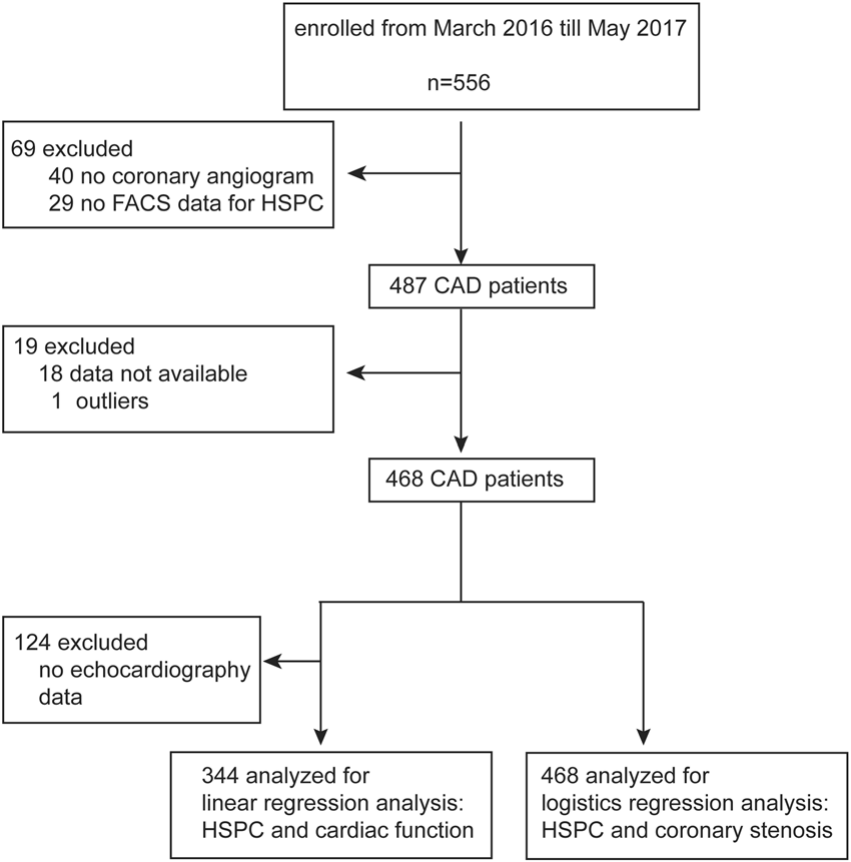
Flowchart. HSPCs, hematopoietic stem/progenitor cells.

From March 2016 to May 2017, 556 patients were enrolled in this study. Flowchart of the participants included in the analysis is shown in Fig. 1. Among the 468 CHD patients, 195 (41.7%) were women. Mean values (±SD) in these 468 participants were 60.9 ± 10.5 years for age, 134.1 ± 17.5 mmHg and 79.8 ± 11.2 mmHg for systolic and diastolic blood pressure, and 4.33 ± 0.99 mmol/L for total cholesterol. Among all participants, 294 (62.8%) had hypertension, of whom 237 (50.6%) were on antihypertensive drugs, and 125 (26.7%) had diabetes.

All patients were examined by coronary angiography for stenoses of four coronary arteries, namely, left main artery, anterior descending branch, left circumflex and right coronary artery. Coronary stenoses was calculated as plaque area divided by lumen area and multiplied by 100. Subjects were diagnosed with CHD if coronary stenoses were equal to or above 50 in any of the coronary arteries based on coronary angiography. Table 1 lists the general characteristics of the subjects with and without CHD.

**Table 1:** Characteristics of the participants.

Characteristic	No CHD	CHD	P value
Number in category	154	314	
**Number of subjects (%)**			
Women	83 (53.9)	112 (35.7)	<0.0001
Smokers	52 (33.8)	165 (52.5)	<0.0001
Drinking alcohol	31 (20.1)	96 (30.6)	0.020
Hypertension	88 (57.1)	206 (65.6)	0.084
Antihypertensive treatment	93 (60.4)	247 (78.7)	<0.0001
dyslipidemia	113 (73.4)	252 (80.3)	0.092
Statins treatment	110 (71.4)	266 (84.7)	0.001
Diabetes mellitus	29 (18.8)	96 (30.6)	0.008
**Mean (SD) of characteristic**			
Age (years)	59.8 (10.1)	61.4 (10.6)	0.10
Systolic pressure (mm Hg)	132.4 (16.2)	134.9 (18.0)	0.13
Diastolic pressure (mm Hg)	79.6 (8.9)	79.9 (12.2)	0.75
Mean arterial pressure (mm Hg)	97.2 (9.7)	98.2 (12.6)	0.31
Heart rate (beats per minute)	71.2 (11.5)	74.8 (12.8)	0.004
**Biochemical data**			
Serum creatinine (μmol/L)	74 (61–84)	74 (64–84)	0.96
eGFR (mL/min/1.73 m^2^)	86.4 (19.0)	87.5 (16.7)	0.53
Plasma glucose (mmol/L)	5.68 (1.40)	6.48 (2.90)	<0.0001
γ-glutamyltransferase (units/L)	22 (17–31)	24 (17–35)	0.10
uric acid (μmol/L)	340.9 (97.3)	331.9 (87.5)	0.31
Serum triglyceride (mmol/L)	1.31 (0.95–1.89)	1.51 (1.07–2.07)	0.17
Total cholesterol (mmol/L)	4.26 (0.94)	4.37 (1.01)	0.26
LDL cholesterol (mmol/L)	2.69 (0.79)	2.82 (0.88)	0.19
HDL cholesterol (mmol/L)	1.09 (0.30)	1.05 (0.24)	0.056
Apolipoprotein A-I (μg/mL)	8.84 (2.09)	8.82 (2.04)	0.95
Apolipoprotein B (μg/mL)	203.0 (46.7)	202.0 (50.1)	0.84
**Inflammatory markers**			
White blood cell count (x10^9^/L)	6.92 (1.7)	7.7 (2.7)	0.001
Neutrophils (x10^9^/L)	4.33 (1.46)	5.13 (2.44)	<0.0001
Lymphocytes (x10^9^/L)	1.91 (0.68)	1.86 (0.77)	0.49
Monocytes (x10^9^/L)	0.47 (0.15)	0.50 (0.20)	0.077
C-reactive protein (mg/L)	0.96 (0.70–1.60)	1.56 (0.89–4.88)	<0.0001
GM-CSF(ng/L)	107.4 (21.7)	108.3 (22.2)	0.70
SDF-1α (pg/mL)	3579.2 (674.9)	3562.3 (648.1)	0.79
**Coagulation markers**			
Fibrinogen (g/L)	3.03 (2.60–3.43)	3.13 (2.69–3.58)	0.19
D-dimer (mg/L)	0.09 (0.06–0.15)	0.11 (0.07–0.18)	0.11
Circulating HSPCs (%)	0.08 (0.03–0.10)	0.10 (0.04–0.12)	0.047

CHD, defined by coronary artery stenosis ≥50% as determined by coronary angiography. HSPCs: hematopoietic stem/progenitor cells. Logarithmically transformed values are expressed as geometric means (interquartile range). Means were compared using *t*-test and proportions by Fisher’s exact test.

**Figure 2:**
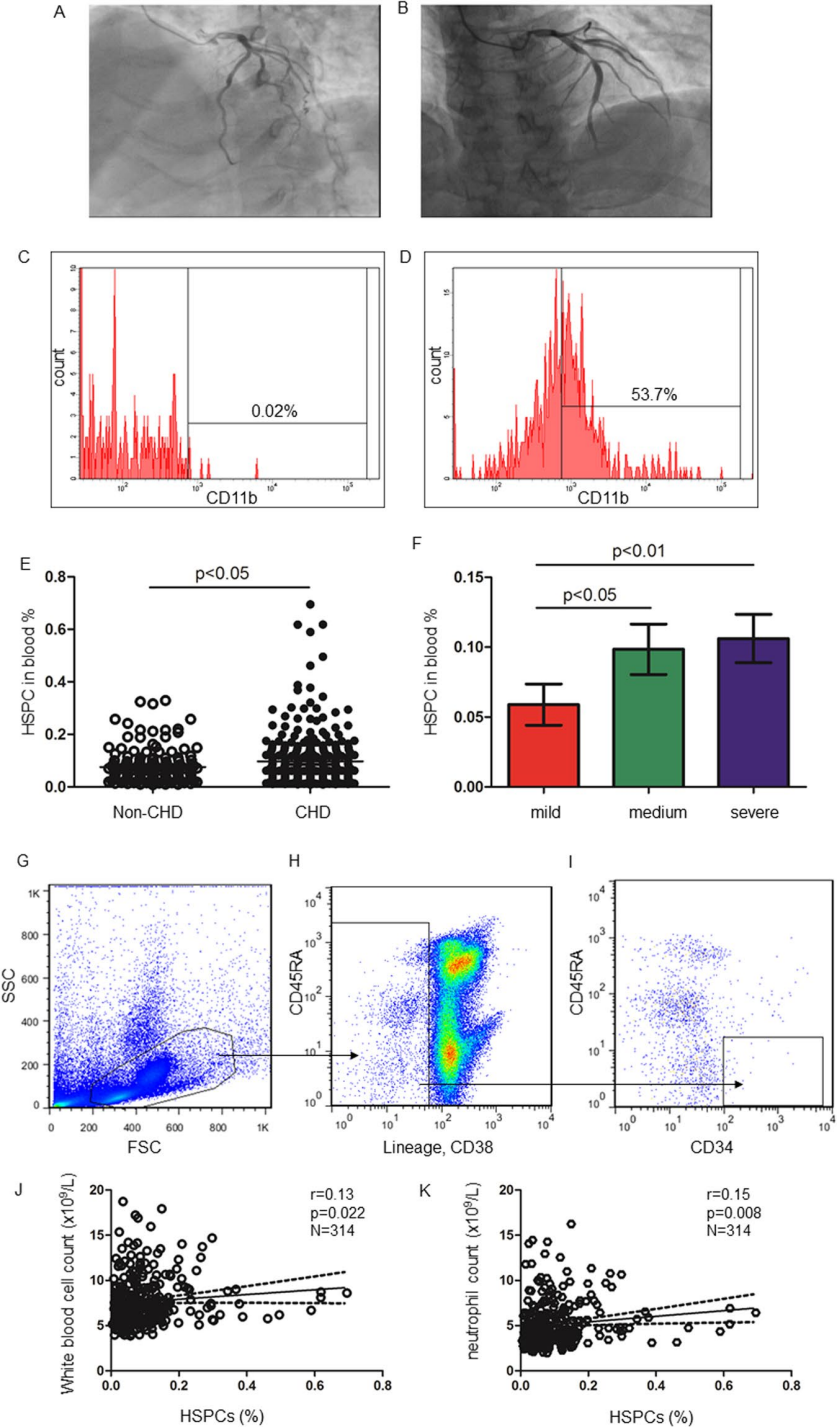
HSPCs frequency in CHD patients. Mild coronary stenosis <50% (**A**) and severe coronary stenosis ≥70% (**B**), as detected by coronary angiography. (**C**) The percentage of CD11b+ cells in freshly isolated HSPCs (**C**). (**D**) HSPCs were plated on Methylcelluose cultures for 14 days. Cells were harvested and stained with anti-human CD11b. The percentage of CD11b+ cells was shown. (**E**) HSPCs frequency in subjects with and without CHD. (**F**) HSPCs frequency in CAD with different extents of stenosis. HSPCs frequencies in e and fare expressed as mean and 95% CI. (**G**–**I**) Representative FACS plot of HSPCs analysis. Among all samples, at least8728 (95%CI, 5783–11674) CD34+ cells were obtained in the analysis. (**J**,**K**) The correlation curves of HSPCs frequency and white blood cell count or neutrophil count in CHD patients, respectively.

Based on the presence of significant stenoses (≥70%), the CHD patients were categorised into three groups: mild, if the stenosis of all coronary arteries was less than 70%; medium, if the stenosis of one of the coronary arteries was equal to or above 70%; and severe, if the stenosis of two or more of the coronary arteries was equal to or above 70% (Fig. 2A,B). Table 2 lists the general characteristics of the subjects by the severity of coronary artery occlusion in CHD patients. Serum LDL-c levels in patients with medium coronary stenosis were higher than those in patients with mild coronary stenosis (Table 2). Although serum levels of GM-CSF, SDF-1α, fibrinogen and D-dimer did not differ among the groups, neutrophil count was greater in patients with medium and severe coronary stenosis than in those with mild stenosis (medium vs. mild: 5.35 ± 2.56 × 10^9^/L vs. 4.38 ± 1.51 × 10^9^/L, p = 0.004; severe vs. mild: 5.15 ± 2.52 × 10^9^/L vs. 4.38 ± 1.51 × 10^9^/L, p = 0.018) (Table 2).

**Table 2:** Characteristics of CHD patients by coronary stenosis severity.

Characteristic	Coronary artery stenoses		
	mild	medium	severe
Number in category	41	132	141
**Number of subjects (%)**			
Women	18 (43.9)	46 (34.8)	48 (34.0)
Smokers	12 (29.3)	77 (58.3)	76 (53.9)
Drinking alcohol	11 (26.8)	44 (33.3)	41 (29.1)
Hypertension	28 (68.3)	84 (63.6)	94 (66.7)
Antihypertensive treatment	34 (82.9)	99 (75.0)	114 (80.9)
Dyslipidemia	113 (73.4)	106 (80.3)	115 (81.6)
Statins treatment	33 (80.5)	114 (86.4)	119 (84.4)
Diabetes mellitus	19 (46.3)	26 (19.7)	51 (36.2)
Myocardial infarction	3 (13.1)	24 (42.9)	29 (51.8)
**Mean (SD) of characteristic**			
Age (years)	62.0 (8.2)	59.6 (10.5)	63.0 (11.1)
Systolic pressure (mm Hg)	138.8 (16.9)	134.4 (19.5)	134.3 (16.8)
Diastolic pressure (mm Hg)	80.9 (11.0)	80.1 (13.0)	79.3 (11.9)
Mean arterial pressure (mm Hg)	100.2 (11.8)	98.2 (13.5)	97.6 (11.9)
Heart rate (beats per minute)	76.0 (12.5)	74.8 (14.4)	74.5 (11.2)
**Biochemical data**			
Serum creatinine (μmol/L)	71 (59–76)	72 (63–83)	78 (66–88)^†^
eGFR (mL/min/1.73 m^2^)	90.8 (13.1)	90.2 (16.1)	83.9 (17.6)^†^
Serum triglyceride (mmol/L)	1.58 (0.92–2.12)	1.57 (1.12–2.18)	1.48 (0.99–1.92)
Total cholesterol (mmol/L)	3.98 (0.76)	4.46 (0.93)^†^	4.40 (1.13)^†^
LDL cholesterol (mmol/L)	2.48 (0.70)	2.88 (0.78)^†^	2.85 (0.98)*
HDL cholesterol (mmol/L)	1.00 (0.22)	1.08 (0.27)	1.03 (0.20)
Fasting blood glucose (mmol/L)	6.74 (2.34)	6.12 (2.46)	6.74 (3.35)
γ-glutamyltransferase (units/L)	21 (15–31)	26 (20–37)	22 (16–32)
uric acid (μmol/L)	304.5 (84.5)	327.4 (82.6)	344.1 (91.2)*
Apolipoprotein A-I (μg/mL)	8.90 (1.85)	8.65 (2.01)	8.97 (2.11)
Apolipoprotein B (μg/mL)	200.9 (45.3)	200.0 (51.7)	204.2 (50.1)
**Inflammation markers**			
HSPC (%)	0.04 (0.03–0.09)	0.07 (0.03–0.13)^†^	0.07 (0.04–0.14)§
C-reactive protein (mg/L)	1.10 (0.79–2.98)	1.56 (0.90–4.59)	1.90 (0.90–6.20)*
GM-CSF (ng/L)	105.5 (21.7)	107.6 (21.9)	109.7 (22.7)
SDF-1α (pg/mL)	3530.7 (612.6)	3553.9 (657.1)	3579.3 (653.6)
WBC count (x10^9^/L)	6.85 (1.91)	7.97 (2.83)^†^	7.68 (2.72)
Neutrophils (x10^9^/L)	4.38 (1.51)	5.35 (2.56)^†^	5.15 (2.52)*
Monocytes (x10^9^/L)	0.48 (0.22)	0.50 (0.20)	0.50 (0.19)
Lymphocytes (x10^9^/L)	1.77 (0.54)	1.92 (0.89)	1.83 (0.71)
**Coagulation markers**			
Fibrinogen (g/L)	2.93 (2.42–3.26)	3.13 (2.73–3.57)	3.20 (2.69–3.70)
D-dimer (mg/L)	0.11 (0.07–0.16)	0.10 (0.06–0.16)	0.11 (0.07–0.19)
Circulating HSPCs (%)	0.06 (0.02–0.07)	0.10 (0.03–0.13)*	0.11 (0.04–0.14)

The extent of CHD was defined by the number of significant coronary artery stenoses. Myocardial infarction included both ST-elevated and non-ST-elevated myocardial infarction. eGFR: estimated glomerular filtration rate. HSPCs: hematopoietic stem/progenitor cells. Logarithmically transformed values are expressed as geometric means (interquartile range). Means were compared using *t*-test and ANOVA and proportions by Fisher’s exact test. Significance of the difference when compared with the mild group: ^*^P ≤ 0.05; ^†^*P* ≤ 0.01.

### Coronary artery stenosis

Human HSPCs are included in the CD34^+^CD38^−^CD45RA^dim^lineage^−^ cell population^16,17^. When CD34^+^CD38^−^CD45RA^dim^lineage^−^ HSPCs were plated for a colony-forming assay and differentiated *in vitro*, they formed myeloid colonies as evidenced by the percentage of CD11b+ cells by FACS analysis (CD11b+ cells% in un-differentiated HSPCs *vs*. differentiated HSPCs: 0.02 ± 0.01% *vs*. 48.67 ± 8.61%, p = 0.012, n = 3 for each) (Fig. 2C,D). FACS analysis illustrated that the proportion of circulating CD34^+^CD38^−^CD45RA^dim^lineage^−^HSPCsamong mononuclear cells was higher in CHD patients than in non-CHD patients (0.10% *vs*.0.08%, p = 0.047) (Table 1). Moreover, the HSPCs frequency was 0.06% in the group with mild coronary stenosis, but increased to 0.10% in the medium and 0.11% in the severe coronary stenosis groups (p < 0.05 for both) (Table 2, Fig. 2E,F). Representative FACS plots showed how HSPCs were analysed by flow cytometry (Fig. 2G–I). By Spearman’s analysis, HSPCs frequency was shown to be positively associated with white blood cell and neutrophil counts in CHD patients (white blood cell count: r = 0.13, p = 0.022; neutrophil count: r = 0.15, p = 0.008) (Fig. 2J,K) but not in non-CHD participants (p ≥ 0.42). By contrast, circulating HSPCs frequency was not associated with differential lymphocyte count in those with or without CHD (p ≥ 0.20).

### Categorical analysis

We first compared the relationship of each marker with the occurrence of CHD. When adjusting for cofounding factors, for a one-SD increase for each of the following variables, the odds ratio of having CHD *vs*. no CHD was 1.24 (95% CI, 1.07–1.48; p = 0.017) for HSPCs, 1.48 (95% CI, 1.23–1.79; p < 0.0001) for CRP, 1.22 (95% CI, 1.01–1.47; p = 0.043) for white blood cell count, 1.26 (95% CI, 1.05–1.53; p = 0.016) for neutrophil count and 1.33 (95% CI, 1.12–1.60; p = 0.002) for low-density lipoprotein cholesterol (LDL-c) (Table 3). After Bonferroni correction for 16 biomarkers, the odds ratio of having no CHD *vs*. CHD remained significantly associated with serum CRP and LDL-c (p < 0.0031).

**Table 3:** Multivariable-adjusted associations of coronary occlusion status with biomarkers.

Coronary occlusion severity	No CHD cs. CHD	CHD	CHD	CHD
	<50% vs ≥50%	Mild vs. medium	Mild vs. severe	Medium vs. severe
**Inflammation markers**				
HSPC (%)	1.24 (1.04 to 1.48)	1.72 (1.17 to 2.54)	2.08 (1.35 to 3.21)^‡^	1.07 (0.83 to 1.38)
C-reactive protein (mg/L)	1.48 (1.23 to 1.79)^§^	1.37 (0.88 to 2.14)	1.67 (1.06 to 2.62)	1.22 (0.94 to 1.58)
GM-CSF (ng/L)	1.07 (0.90 to 1.27)	1.09 (0.72 to 1.65)	1.33 (0.92 to 1.96)	1.12 (0.88 to 1.45)
SDF-1α (pg/mL)	1.00 (1.00 to 1.00)	1.00 (0.52 to 1.93)	1.00 (1.00 to 1.00)	1.00 (1.00 to 1.00)
White blood cells	1.22 (1.01 to 1.47)	1.57 (0.94 to 2.63)	1.38 (0.86 to 2.21)	0.91 (0.70 to 1.17)
Neutrophils (x10^9^/L)	1.27 (1.04 to 1.53)	1.62 (0.94 to 2.80)	1.47 (0.88 to 2.44)	0.93 (0.73 to 1.18)
Monocytes (x10^9^/L)	1.06 (0.88 to 1.27)	0.99 (0.99 to 1.00)	0.89 (0.61 to 1.32)	1.04 (0.81 to 1.34)
Lymphocytes (x10^9^/L)	0.97 (0.81 to 1.16)	1.14 (0.77 to 1.70)	1.03 (0.65 to 1.62)	0.93 (0.73 to 1.20)
**Coagulation markers**				
Fibrinogen (g/L)	0.95 (0.80 to 1.13)	1.07 (0.74 to 1.55)	0.99 (0.64 to 1.52)	1.04 (0.78 to 1.39)
D-dimer (mg/L)	1.13 (0.95 to 1.35)	1.03 (0.75 to 1.55)	0.92 (0.64 to 1.33)	1.04 (0.79 to 1.37)
**Lipids and metabolism markers**				
Serum triglyceride (mmol/L)	1.02 (0.85 to 1.23)	1.24 (0.77 to 2.01)	0.94 (0.70 to 1.26)	0.74 (0.54 to 1.01)
LDL-cholesterol (mmol/L)	1.33 (1.12 to 1.60)^†^	2.09 (1.20 to 3.62)	1.67 (1.11 to 2.51)	1.00 (0.70 to 1.43)
HDL-cholesterol (mmol/L)	0.91 (0.76 to 1.09)	1.65 (1.03 to 2.64)	1.34 (0.78 to 2.28)	0.80 (0.57 to 1.12)
Apolipoprotein A-1 (μg/mL)	1.06 (0.89 to 1.26)	0.98 (0.65 to 1.47)	0.95 (0.64 to 1.40)	1.16 (0.90 to 1.51)
Apolipoprotein B (μg/mL)	0.95 (0.82 to 1.16)	1.05 (0.71 to 1.55)	1.05 (0.71 to1.55)	1.00 (0.78 to 1.28)
Uric acid (μmol/L)	1.00 (0.83 to 1.27)	1.10 (0.69 to 1.72)	1.44 (0.91 to 2.26)	1.31 (0.91 to 1.72)

All analyses were adjusted for covariables, including age, sex, mean arterial pressure, heart rate, plasma glucose, serum creatinine, plasma glucose, γ-glutamyltransferase, smoking, alcohol intake, history of hypertension (1 or 0), history of diabetes (1 or 0), use of diuretics, inhibitors of the renin–angiotensin system (β-blockers, angiotensin-converting-enzyme inhibitors and angiotensin type-1 receptor blockers), vasodilators (calcium-channel blockers and α-blockers), metformin and statins. With the exceptions of LDL-c and HDL-c, all other biomarkers were additionally adjusted with the total-to-HDL cholesterol ratio. A value of *P* < 0.0031 was considered significant after the Bonferroni correction. Significance of the associations: ^†^*P* < 0.01; ^‡^*P* < 0.001; ^§^*P* ≤ 0.0001.

Among CHD patients, for a one-SD increase in each of the following variables, the multivariable-adjusted odds ratios expressing the risk of having medium coronary artery stenosis ≥70% *vs*. 50*–*70% were 1.72 (95% CI, 1.17–2.54; p = 0.006) for HSPCs, 1.36 (95% CI, 0.88–2.14, p = 0.17) for CRP, 1.57 (95% CI, 0.94–2.63, p = 0.09) for white blood cell count, 1.62 (95% CI, 0.94–2.80; p = 0.10) for neutrophil count and 2.08 (95% CI, 1.20–3.62; p = 0.023) for LDL-c (Table 3). Similarly, multivariate-adjusted logistic analysis demonstrated that, for a one-SD increase, the odds ratios expressing the risk of having severe coronary stenosis ≥70% vs. mild coronary stenosis <70% were 2.08 (95% CI, 1.35–3.21; p = 0.0009) for HSPCs, 1.67 (95% CI, 1.06–2.62, p = 0.026) for CRP, 2.45 (95% CI, 0.86–2.21, p = 0.18) for white blood cell count, 1.47 (95% CI, 0.88–2.44; p = 0.15) for neutrophil count and 1.67 (95% CI, 1.11–2.51, p = 0.014) for LDL-c (Table 3). Nevertheless, neither HSPCs frequency nor other markers were associated with the risk of having medium *vs*. severe stenosis (p ≥ 0.05) (Table 3). After Bonferroni correction, HSPCs was the only marker that was associated with the odds ratio of having mild *vs*. severe coronary stenosis (p < 0.0031) whereas none of these markers was associated with the odds ratio of having mild *vs*. medium coronary stenosis.

**Figure 3:**
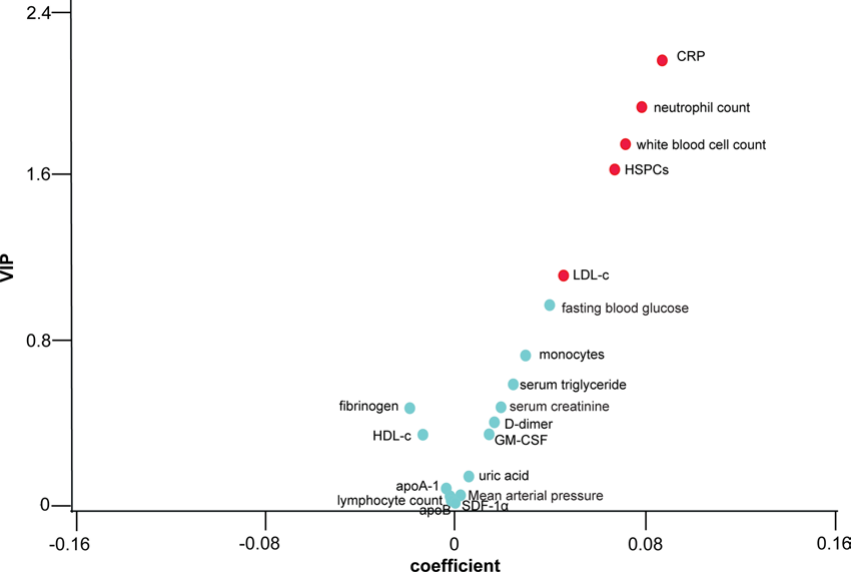
We divided the study population of 468 participants using coronary artery stenosis and circulating biomarkers. V-plots were generated for the PLS-DA-derived VIP scores versus the centred and rescaled correlation coefficients. HSPCs, neutrophils, lymphocytes and monocytes are expressed as percentages among white blood cells. HSPCs, CRP, serum triglyceride, fibrinogen and D-dimer were logarithmically transformed in the analysis. VIP represents the importance of each marker in the construction of the PLS factors. The correlation coefficients reflect the association of significant coronary stenosis (≥70%) with each marker. The markers with VIP score ≥1 and VIP score <1 are indicated in red and cyan blue, respectively.

When we pooled non-CHD subjects and CHD patients together, the V-plot confirmed the results of logistic analyses (Table 3) by showing that HSPCs, CRP, white blood cell count, neutrophil count and LDL-c were associated with significant coronary stenosis ≥70% vs. <70% (Fig. 3).

### ROC

**Figure 4:**
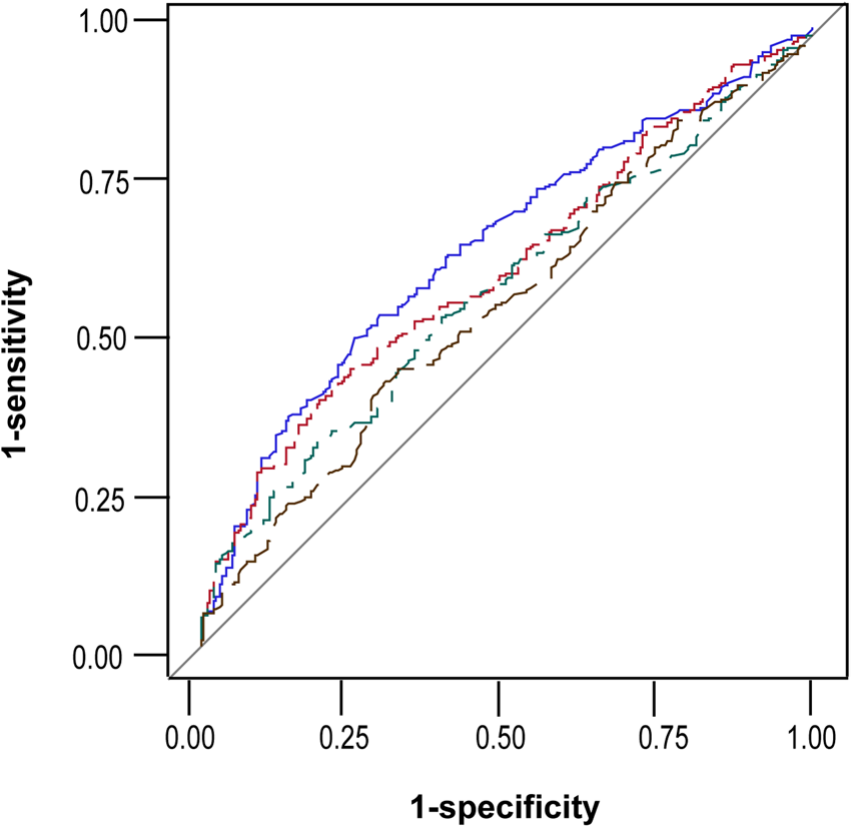
Receiver operating characteristic (ROC) curves for prediction of significant coronary stenosis (from <70% to ≥70%). Blue, red, green and black lines identify C-reactive protein, HSPCs, white blood cell count and LDL-cholesterol, respectively.

CRP is a general inflammatory marker related to inflamed atherosclerotic plaque. The area under the curve (AUC) for CRP in the discrimination between stenosis <70% and ≥70% was 0.64 (95% CI, 0.59–0.69) in all subjects. Using CRP as a reference (Fig. 4), the AUC values of HSPCs, white blood cell count and LDL-c were 0.60 (95% CI, 0.54–0.65; p = 0.21), 0.58 (95% CI, 0.53–0.64; p = 0.072) and 0.56 (95% CI, 0.51–0.61, p = 0.022), respectively. These data suggest that HSPCs, white blood cell count and CRP were equally potent at discriminating significant coronary stenosis.

### Cardiac function by echocardiography

Finally, we analysed the relationship between these biomarkers and cardiac function. Among all subjects, 344 were examined by echocardiography. Left ventricular ejection fraction, end-systolic diameter and end-diastolic diameter did not differ between non-CHD subjects and CHD patients (Table 4).

**Table 4:** Characteristics of the study participants.

Characteristic	Non-CHD	CHD	P value
Number in category	92	252	
Women (%)	52 (56.5%)	95 (37.7%)	
**Mean (SD) of characteristic**			
Left ventricular post wall (mm)	9.77 (1.51)	10.19 (1.69)	0.062
Left ventricular diastolic diameter (mm)	49.26 (4.55)	49.72 (5.60)	0.439
Left ventricular systolic diameter (mm)	30.42 (5.31)	31.12 (6.44)	0.354
Movement range of interventricular septal (mm)	9.08 (3.42)	8.09 (2.59)	0.010
Movement range of left ventricular post wall (mm)	11.34 (2.36)	11.12 (2.41)	0.517
Ejection fraction (%)	68.22 (8.97)	67.08 (10.31)	0.347

CHD, coronary heart disease. Data are expressed as mean (SD). Means were compared using *t*-test between two groups.

Multivariate-adjusted linear regression analysis demonstrated that one-SD increases of circulating HSPCs frequency, serum CRP level, white blood cell count and neutrophil count were associated with 2.33% (95% CI, −4.61% to −0.06%; p = 0.045), 3.04% (95% CI, −5.65% to −0.44%; p = 0.023), 1.92% (95% CI, −3.28% to −0.56%; p = 0.007) and 2.25% (95% CI, −3.58% to −0.09%; p = 0.01) declines of left ventricular ejection fraction in CHD patients, respectively. Furthermore, one-SD increases of circulating HSPCs frequency, white blood cell count and neutrophil count were associated with 2.42 mm (95% CI, 0.03–2.93 mm; p = 0.045), 1.12 mm (0.23–2.01 mm; p = 0.013) and 1.23 mm (95% CI, 0.38–2.09 mm; p = 0.004) increases of end-systolic diameter in the left ventricle in CHD patients, respectively. By contrast, CRP was not associated with end-systolic diameter in the left ventricle (p = 0.26). Moreover, LDL-c was associated with neither ejection fraction nor end-systolic diameter (p ≥ 0.19). Bonferroni correction of 16 biomarkers did not identify any significant association between any biomarker and cardiac function studied.

**Figure 5:**
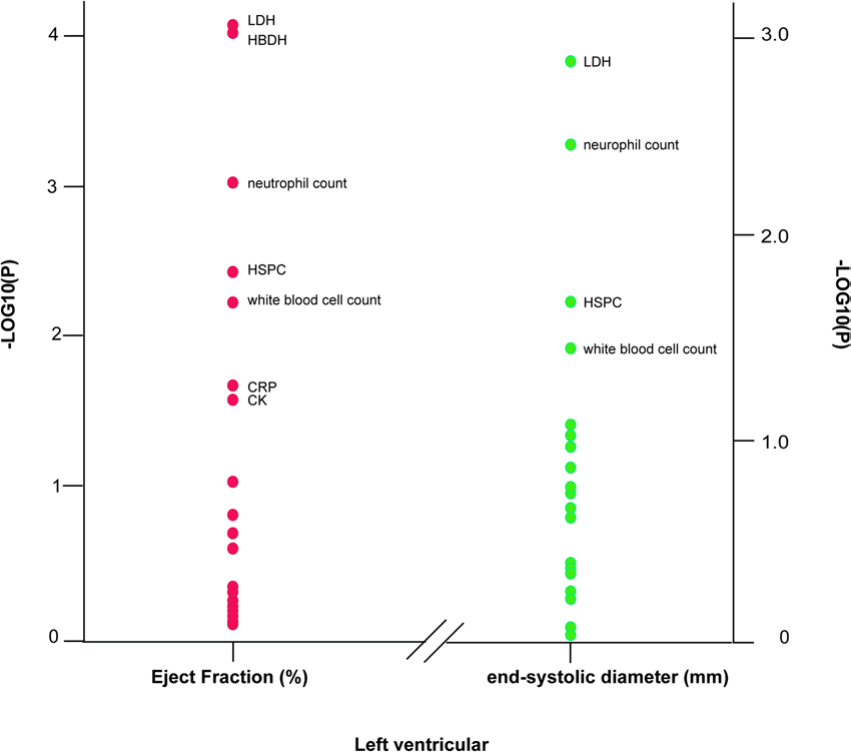
−Log10(p) probability plot of the multivariable-adjusted associations of various indexes of left ventricular function with the biomarkers. −Log10(p) probability plot of the multivariable-adjusted associations of various indexes of left ventricular function with the biomarkers in CHD patients. All analyses were adjusted for covariables, including age, sex, mean arterial pressure, heart rate, plasma glucose, serum creatinine, plasma glucose, γ-glutamyltransferase, smoking, alcohol intake, history of hypertension (1 or 0), history of diabetes (1 or 0), use of diuretics, inhibitors of the renin–angiotensin system (β-blockers, angiotensin-converting-enzyme inhibitors and angiotensin type1 receptor blockers), vasodilators (calcium-channel blockers and α-blockers), metformin and statins. With the exceptions of LDL-c and HDL-c, all other biomarkers were additionally adjusted with total-to-HDL-c ratio. Biomarkers in Fig. 3 as well as creatinine kinase (CK), brain natriuretic peptide (NT-pro-BNP), lactic dehydrogenase (LDH), α-hydroxybutyrate dehydrogenase (HBDH) and cardiac troponin I (TnI) were analysed and those with p < 0.05 are named here.

Besides the biomarkers mentioned above, conventional cardiac injury markers including serum creatinine, brain natriuretic peptide (NT-pro-BNP), lactic dehydrogenase (LDH), α-hydroxybutyrate dehydrogenase (HBDH) and cardiac troponin I (TnI) were included individually in the analysis^18,19^. Serum creatinine and serum lactic dehydrogenase were negatively associated with ejection fraction and positively associated with end-systolic diameter of the left ventricle in CHD patients. Nevertheless, none of the biomarkers was associated with end-dilation diameter of the left ventricle in the patients after adjusting for covariables (p ≥ 0.12). Figure 5 shows the −log10(*P*) probability plot of the multivariable-adjusted association of various markers with ejection fraction (continuous) or end-systolic diameter (continuous) of the left ventricle in CHD patients.

## Discussion

The key findings of this study can be summarised as follows. (1) HSPCs frequency in the peripheral blood was 1.8-fold higher in CHD patients than in non-CHD participants. (2) By multivariate-adjusted logistic analysis, per one-SD increase for each of the following variables, the odds ratio of having CHD *vs*. not having it was 1.24 for HSPCs (p = 0.017), 1.48 for CRP (p < 0.0001), 1.22 for white blood cell count (p = 0.043) and 1.33 for LDL-c (p = 0.002). (3) Likewise, in CHD patients, per one-SD increase for each of the following variables, the odds ratio of having one coronary artery stenosis ≥70% vs. <70% was 1.72 for HSPCs (p = 0.006), 1.37 for CRP (p = 0.17), 1.56 for white blood cell count (p = 0.09) and 2.08 for LDL-c (p = 0.009). For CHD patients, per one-SD increase of each variable, the odds ratio of having mild vs. severe coronary stenosis was 2.08 for HSPCs (p = 0.0009), 1.67 for CRP (p = 0.026), and 1.67 for LDL-c (p = 0.014). (4) HSPCs frequency, white blood cell count and CRP level discriminated coronary occlusion <70% vs. ≥70% with comparable potency. (5) By multivariate-adjusted linear regression analysis, circulating HSPCs frequency, white blood cell count and neutrophil count were inversely correlated with left ventricular ejection fraction and positively correlated with end-systolic diameter of the left ventricle in CHD patients. CRP was negatively associated with ejection fraction but not related to end-systolic diameter of the left ventricle in the patients. And (6) After Bonferroni correction for 16 biomarkers, CRP and HSPCs remained significantly associated with the odd ratio of having CHD *vs*. no CHD and mild *vs*. severe coronary stenosis, respectively.

Atherosclerosis is the underlying pathogenesis of CHD and contributes substantially to cardiac dysfunction and disease progression. Inflammatory cells and pro-inflammatory cytokine and chemokine production from inflammatory cells are hallmarks of atherosclerotic plaque^10–12^. The evolution of early/stable plaque to advanced/vulnerable plaque results from a phenotypic transition from controlled to uncontrolled inflammation^3^. HSPCs give rise to all types of blood cells and are the source of inflammatory cells. They could thus be an important biomarker and intervention target for atherosclerosis.

This study on CHD patients was prompted in part by previous observations that hypercholesterolemia induced by a high-fat diet enhanced the proliferation of HSPCs and their subsequent differentiation to myeloid cells, including neutrophils, whereas the control of HSPCs proliferation and differentiation reversed hypercholesterolemia-induced leukocytosis and reduced atherosclerosis plaque in mice^13,15^. In this study, we reported that circulating HSPCs frequency was tightly associated with coronary stenoses in CHD patients. Compared with CRP, the conventional biomarker of coronary stenosis^20^, HSPCs is more sensitive in assessing the progression from mild stenosis (<70%) to medium stenosis (≥70%) and reflecting left ventricular end-systolic diameter in CHD patients. We further applied a correction for multiple testing for all the biomarkers studied^21^. By Bonferroni correction, HSPCs remained the only marker that was associated with odds ratio of having mild *vs*. severe coronary stenosis in CHD patients.

GM-CSF and SDF-1α trigger HSPCs mobilisation from bone marrow to peripheral blood^22–24^. Moreover, a positive correlation between serum levels of SDF-1αand coronary artery occlusion was reported in CHD patients^25^. In parallel with this, when atheroma cell suspension was obtained from carotid artery, GM-CSF levels were higher in patients with symptomatic plaque than in those with asymptomatic plaque^26^. In this study, we performed a detailed dissection of the relationships between GM-CSF, SDF-1α, HSPCs and coronary stenosis. Circulating HSPCs frequency was found to be positively associated with serum levels of GM-CSF (r = 0.12, p = 0.011) but not with SDF-1α (r = 0.02, p = 0.61). Unlike HSPCs, neither GM-CSF nor SDF-1αwas associated with the severity of coronary stenosis in CHD patients. These findings indicate that GM-CSF partially contributed to the increase in HSPCs in the blood. GM-CSF and SDF-1α appear to have weak effects on coronary occlusion.

Decreasing LDL-c and elevating HDL-c have been therapeutic targets for attenuating atherosclerosis and treating ischemic heart disease for decades. Recently, meta-analysis of multiple statin trials elegantly demonstrated the beneficial effects of statins in lowering LDL-c and reducing cardiovascular risks^27,28^. Proprotein convertase subtilisin-kexin type 9 (PCSK9) inhibitors are another drug family that can reduce LDL-c. Inhibition of PCSK9 abrogates LDL-receptor degradation and thus enhances LDL uptake for clearance^29^. PCSK9 inhibitors have also been shown to lead to a 60% reduction in LDL-c without any indication of side effects^30,31^. In the current study, we illustrated that LDL-c was increased in CHD patients and positively associated with atherosclerosis-based coronary stenosis even after adjusting for statins and other potential covariables. Our data are in line with other studies supporting the concept that LDL-c is an appropriate target for cardiovascular disease treatment. Paradoxically, a Mendelian randomisation study involving 111,194 individuals from two prospective general populations reported that a low LDL-c level due to genetic variation in *PCSK9* and *HMGCR* is associated with a high risk of neurocognitive diseases including Alzheimer’s disease and Parkinson’s disease^32^. Therefore, there is a need for further investigation of the optimal therapeutic range of LDL-c for treating cardiovascular disease and protecting against adverse neurocognitive events.

Atherothrombosis is another major cause of coronary occlusion. D-dimer is a product of the degradation of cross-linked fibrin and is thus commonly used as a marker to predict plaque severity based on the Gensini score^33^. In the current study, despite the extensive administration of anti-coagulation drugs, serum D-dimer and fibrinogen did not differ between subjects with and without CHD. In addition, none of these variables had any significant association with the severity of coronary stenosis in CHD patients.

The present study should be interpreted in consideration of its limitations. First, this is a cross-sectional study. Whether HSPCs could predict the outcome of adverse cardiovascular events or the incidence of CHD remains to be proven in longitudinal studies. Recently, Hammadah measured CD34^+^ cells in CHD patients and found that their low levels in circulation independently predict adverse cardiovascular disease outcomes^34^. A follow-up study of these patients would provide a better understanding of the role of HSPCs in cardiovascular disease outcomes. Second, we did not categorise monocytes into M1 and M2 subtypes or other subgroups. Third, we could not rule out the possibility that the increased HSPCs frequency in peripheral blood was derived from increased HSPCs proliferation or mobilisation from bone marrow into circulation. Fourth, we did not have data on the body mass of the subjects because, when most of them arrived, it was an emergency situation. As we included extensive covariables for adjustment, the impact of body mass index on the analysis should have been limited.

In conclusion, we identified HSPCs as an important marker to assess atherosclerosis-induced coronary stenosis. The level of circulating HSPCs increases in association with the occurrence of CHD and is significantly associated with the progression of mild coronary occlusion to a severe state. The increase of HSPCs in CHD patients has an adverse impact on ejection fraction and is positively associated with end-systolic diameter in the left ventricle. Further studies are required to testify whether HSPCs could be as a novel intervention target for CHD patients.

## Methods

### Subjects

All study procedures complied with the Declaration of Helsinki regarding investigations of human subjects. They received ethical approval from the institutional review boards of both Lu He Hospital and Capital Medical University. All participants provided written informed consent.

From March 2016 to May 2017, 556 patients were enrolled in this study. Their blood pressure was recorded as the mean of three readings and the mean arterial pressure was determined as diastolic pressure plus one-third of pulse pressure. Hypertension was defined as blood pressure of at least 140 mmHg systolic or 90 mmHg diastolic or the use of antihypertensive drugs. Diabetes was defined as plasma glucose of at least 7.0 mmol/L while fasting or of 11.0 mmol/L or more 2 h after an orally administered glucose load of 75 g. Additional characteristics including age, medical history, smoking and drinking habits, and intake of medications were also recorded.

We excluded 88 patients because of no coronary angiography having been performed (n = 40), lack of FACS-based HSPC data (n = 29), missing basic information (n = 18) or values exceeding the mean by three standard deviations (SDs) or more (n = 1). Thus, in total, 468 participants were statistically analysed. Among these CAD patients, 344 were examined by echocardiography. A flowchart of the study is presented in Fig. 1.

### Echocardiography

Echocardiography was performed prior to coronary artery angiography. A single observer performed the echocardiography using a Philips iE33 (Philips, Amsterdam, Netherlands) device and analysed the digitally stored images, averaging three heart cycles, using a workstation running Hina Uses Workstation (version 2.0; Hina, China). Analyses of the echocardiography images were performed by an investigator who was blinded to the identity of the specific groups. Briefly, diastolic left ventricular (LV) function included the peak early (E) and late (A) diastolic velocities and flow duration from the transmitral blood flow Doppler signal, together with left ventricular ejection fraction (LVEF), left ventricular end-diastolic diameter, left ventricular end-systolic diameter, interventricular septal thickness, ventricular septal amplitude, and left ventricular volume including end-systolic volume (ESV) and end-diastolic volume (EDV). LVEF was calculated as follows:

$${\rm{LVEF}}=(\mathrm{EDV}-\mathrm{ESV})/\mathrm{EDV}\times 100 \% .$$

### Coronary angiography

The patients were examined by computed tomography (CT)-based coronary angiography in accordance with standard criteria (Philips FD10, the Netherlands). The stenoses in the left main coronary artery, anterior descending branch, left circumflex and right coronary artery were evaluated by two experienced cardiologists who were blinded to the group randomisation. In cases of disagreement, consensus was reached by further joint reading.

### Biochemical measurement

After overnight fasting, venous blood samples were drawn for measurement of the total and differential white blood cell counts, serum total cholesterol, HDL cholesterol, triglycerides, creatinine, D-dimer, C-reactive protein (CRP), plasma glucose and γ-glutamyltranspeptidase. Estimated glomerular filtration rate (eGFR) was derived from serum creatinine using the Chronic Kidney Disease Epidemiology Collaboration (CKD-EPI) equation^35^. LDL cholesterol (LDL-c) was computed from serum total and HDL cholesterol (HDL-c) and serum triglycerides by the Friedewald equation^36^. Participants were classified as having dyslipidaemia if at least one of the following criteria was met: total cholesterol higher than 4.9 mmol/L, LDL cholesterol exceeding 3 mmol/L, triglycerides higher than 1.7 mmol/L or HDL cholesterol less than 1.2 mmol/L in women and 1 mmol/L in men^37^. Serum creatinine, high-sensitive C-reactive protein (CRP), cardiac creatinine, lactic dehydrogenase (LDH), α-hydroxybutyrate dehydrogenase (HBDH) and cardiac troponin I (TnI) were measured in the central laboratory at Lu He Hospital.

### Colony-forming assays

Mononuclear cells in peripheral blood were stained with an antibody cocktail against lineage, CD34, CD45RA and CD38. Lin^−^CD34^+^CD38^−^CD45RA^dim^ cells were sorted by FACS and plated with Methocult H4434 (Stem Cell Technologies, Vancouver, Canada). After 10–14 days, colonies were assessed under a light microscope. To confirm the lineages of cells in generated colonies, cells were harvested and stained with anti-human CD11b for FACS analysis.

### Flow cytometry

HSPCs constitute a tiny population among white blood cells. Therefore, to achieve reliable analysis, mononuclear cells were isolated from peripheral blood by Ficoll-based density gradient centrifugation, as described previously^16^. After isolation, cells were stained with an antibody cocktail containing anti-human lineage cocktail APC (BD), anti-human CD38 APC (eBioscience), anti-human CD34PE (eBioscience) and anti-human CD45RA PerCP-Cy5.5 (eBioscience). Dead cells were excluded by their size forward scatter (FSC) and side scatter (SSC) and staining of 7-AAD. Cells stained with isotype antibodies or unstained cells were used as a negative control for FACS analysis. At least 100,000 events were acquired for FACS analysis. Data were acquired using a FACS Canto (BD) and analysed by FlowJo. HSPCs, defined as Lin^−^CD34^+^CD38^−^CD45RA^dim^ cells, were counted. The frequency of HSPCs among mononuclear cells was calculated.

### Enzyme-linked immunosorbent assay

The concentrations of serum N-terminal pro-B-type natriuretic peptide (NT-pro-BNP), stromal-derived factor 1α (SDF-1α), granulocyte-macrophage colony stimulating factor (GM-CSF), apolipoprotein A-1 (apoA-1), apolipoprotein B (apoB) and D-dimer were measured by ELISA, in accordance with the manufacturer’s instructions (MLBio, Shanghai, China). Intra-assay coefficients of variation for NT-pro-BNP, SDF-1a, GM-CSF, apoA-1 and apoB were 0.25%, 0.29%, 0.48%, 0.27% and 0.59%, while inter-assay ones were 1.99%, 4.93%, 4.81%, 3.13% and 3.73%, respectively.

### Statistical analysis

For database management and statistical analysis, we used the SAS system, version 9.4 (SAS Institute Inc., Cary, NC, USA). We normalised the distributions of γ-glutamyltransferase, HSPC, serum creatinine, serum triglyceride, fibrinogen and D-dimer by logarithmic transformation. Means were compared using *t*-test or ANOVA and proportions by Fisher’s exact test. Pearson’s correlation was used for single association analysis. For *t*-test, ANOVA, proportions by Fisher’s exact test and Pearson’s correlation, a p-value < 0.05 was considered statistically significant.

Subjects with coronary artery stenosis ≥50% were diagnosed as having CHD. The extent of CHD was further categorised into mild, intermediate and severe groups using the following criteria: all stenosis <70%, one coronary artery stenosis ≥70% or more than one coronary artery stenosis ≥70%, respectively. Thereafter, multivariate-adjusted logistic analysis was performed to evaluate the coronary occlusion severity with each marker independently. The importance of each marker with its association to coronary stenoses(≥70% vs. <70%)) was assessed from the Variable Importance in Projection (VIP) scores of Wold in all study subjects following construction of the partial least square (PLS) factors. We further evaluated the potential of CRP, circulating HSPCs, white blood cell count and LDL-c to discriminate between CHD patients and controls with coronary stenosis ≥70% vs. <70% by constructing receiver operating characteristic (ROC) curves and by calculating the area under them (AUC). The 95% confidence interval (95% CI) of the AUC was calculated by the DeLong method.

Multivariate-adjusted linear regression analysis was performed for the regression of LV function against each marker in CHD patients. While accounting for covariables, we performed regression of the indexes of diastolic LV function on the markers and constructed –log10 probability plots. Bonferroni correction was applied for variants (i.e. biomarkers) that were associated with coronary stenosis or cardiac function index. A P value < 0.0031 (0.05/16) was considered statistically significant. In this study, all analyses included adjustments for covariables, including age, sex, mean arterial pressure, heart rate, plasma glucose, serum creatinine, plasma glucose, γ-glutamyltransferase, smoking, alcohol intake, history of hypertension (1 or 0), history of diabetes (1 or 0), use of diuretics, inhibitors of the renin–angiotensin system (β-blockers, angiotensin-converting-enzyme inhibitors and angiotensin type-1 receptor blockers), vasodilators (calcium-channel blockers and α-blockers), metformin and statins. With the exceptions of LDL-c and HDL-c, all other biomarkers were additionally adjusted for using the total-to-HDL-cholesterol ratio.

## Data Availability

The corresponding author will make anonymized data available to researchers who present an outstanding research plan that will move the field forward.
